# Nitrosative stress induces DNA strand breaks but not caspase mediated apoptosis in a lung cancer cell line

**DOI:** 10.1186/1477-3163-3-16

**Published:** 2004-12-23

**Authors:** Brandon G Bentz, Neal D Hammer, James A Radosevich, G Kenneth Haines

**Affiliations:** 1 Department of Surgery, Division of Otolaryngology-Head and Neck Surgery, University of Utah, 3362 Huntsman Cancer Institute, 2000 Circle of Hope, Salt Lake City, UT, 84112-5550, USA; 2 Center for Molecular Biology of Oral Disease (MC860), College of Dentistry, University of Illinois at Chicago, USA and the Jesse Brown VAMC, 801 South Paulina Street, Chicago, IL, 60612-7213, USA; 3 Department of Pathology, Northwestern University Medical Center, W127 Ward 6-223, 303 East Chicago, Ave., Chicago, IL, 60611, USA

**Keywords:** Nitric Oxide, DNA Strand Breaks

## Abstract

**Background:**

Key steps crucial to the process of tumor progression are genomic instability and escape from apoptosis. Nitric oxide and its interrelated reactive intermediates (collectively denoted as NO_X_) have been implicated in DNA damage and mutational events leading to cancer development, while also being implicated in the inhibition of apoptosis through S-nitrosation of key apoptotic enzymes. The purpose of this study was to explore the interrelationship between NO_X_-mediated DNA strand breaks (DSBs) and apoptosis in cultured tumor cell lines.

**Methods:**

Two well-characterized cell lines were exposed to increasing concentrations of exogenous NO_X _via donor compounds. Production of NO_X _was quantified by the Greiss reaction and spectrophotometery, and confirmed by nitrotyrosine immunostaining. DSBs were measured by the alkaline single-cell gel electrophoresis assay (the COMET assay), and correlated with cell viability by the MTT assay. Apoptosis was analyzed both by TUNEL staining and Annexin V/propidium iodine FACS. Finally, caspase enzymatic activity was measured using an in-vitro fluorogenic caspase assay.

**Results:**

Increases in DNA strand breaks in our tumor cells, but not in control fibroblasts, correlated with the concentration as well as rate of release of exogenously administered NO_X_. This increase in DSBs did not correlate with an increase in cell death or apoptosis in our tumor cell line. Finally, this lack of apoptosis was found to correlate with inhibition of caspase activity upon exposure to thiol- but not NONOate-based NO_X _donor compounds.

**Conclusions:**

Genotoxicity appears to be highly interrelated with both the concentration and kinetic delivery of NO_X_. Moreover, alterations in cell apoptosis can be seen as a consequence of the explicit mechanisms of NO_X _delivery. These findings lend credence to the hypothesis that NO_X _may play an important role in tumor progression, and underscores potential pitfalls which should be considered when developing NO_X_-based chemotherapeutic agents.

## Background

Nitric oxide (NO^•^) is a ubiquitous nitrogen radical species that has been found to exert protean influences on physiologic and pathophysiologic processes in a wide variety of organ systems. Vastly increasing the functional consequences of NO^• ^production is its interrelationship between the nitroxyl anion (NO^-^) and the nitrosonium cation (NO^+^) depending upon the redox environment in which NO^• ^is being produced. Each of these interrelated redox species demonstrates its own biological consequences. Collectively, nitric oxide biology attributable to the integrated actions of these three species (referred to as NO_X_) demonstrates broad reaching consequences.

It is generally thought that well regulated levels of NO_X _production is important to numerous physiologic processes, while NO_X _overproduction is increasingly being implicated in pathophysiologic processes via nitrosative stress. One molecular mechanism underlying these pathophysiologic processes is NO_X_-mediated genomic damage inducing apoptosis in susceptible cells [1]. This induction of apoptosis is thought to be dependent upon an intact p53-pathway in response to genotoxicity [2].

While the field of NO_X _in cancer biology is a new and developing area of research, the intricacies of NO_X _influence are beginning to be elucidated. We have previously demonstrated widespread increases in expression of nitric oxide synthase isoforms within human primary tumors when compared to surrounding normal tissues [3-6]. Both cancer promotive and cancer protective roles have been ascribed to NO_X_. Cancer promoting effects include: 1) the capacity to trigger mutagenesis, 2) enhance growth, invasion, angiogenesis and metastasis of tumors, 3) select for increasingly virulent tumor cell clones, and 4) suppress the host anti-tumor immune response [7,8]. Cancer protective actions are manifest in the ability of immune-mediated NO_X _production to attenuate tumor cell respiration and DNA synthesis as well as to trigger apoptosis. The mechanistic basis of these differences remains unknown, but may be explained by: 1) differences in the relevant levels of NO_X_-species, 2) the redox environment of NO_X _production, 3) the presence and proximity of downstream response elements within cells under study, 4) the susceptibility of these NO_X _targets, 5) as well as the rate of NO_X _detoxification.

If DNA repair mechanisms are overwhelmed or are incapable of adequate repair, endogenous cell death is triggered via apoptosis. This "programmed cell death" thus prevents damaged cells from undergoing replication perpetuating mutational events. Chronic nitrosative stress is implicated in inhibiting apoptosis. Potential mechanisms for this inhibition of apoptosis include the alteration of protein transcription and translation, or post-translational control of protein function [9]. NO_X _can alter protein function through post-translational modification of thiol groups via S-nitrosation, a key motif within many enzymes and structural proteins [10]. One target of this type of post-translational modulation of apoptotic enzymatic activity are the caspases [11].

Thus, nitrosative stress can on one hand induce DNA damage while inhibiting apoptosis, on the other. This lends mechanistic support to the hypothesis that chronic nitrosative stress may be cancer promotive in specific situations. Based on these data, the purpose of this study was to investigate whether cultured tumor cells exposed to exogenously administered nitrosative stress undergo NO_X_-mediated DNA damage. Furthermore, we investigate the relationship between this ongoing DNA damage and cell death via apoptosis within our cell lines of interest.

## Methods

### Cell Lines

The human lung adenocarcinoma cell line (A549) and the control SV40 transformed human fibroblast cell line (WI38) were obtained from the American Type Culture Collection (Manassas, VA). A549 was grown in RPMI media, while WI38 cells were grown in MEM (Gibco, Paisley, UK). All media was supplemented with 10% fetal calf serum, penicillin, streptomycin, L-glutamine, and fungizone. For various tests, cells were harvested after trypsin-EDTA treatment, washed with Dulbecco's PBS, and resuspended in serumless media.

### Chemicals

Cells were incubated with one of four NO_X_-donor compounds that differed in their mode of donation of NO-equivalents and their half-lives. Two of these compounds were NONOate donor compounds [diethylenetriamine-NONOate (DETA-NONOate, Sigma Chemical Company, St. Louis, MO) and Spermine-NONOate (Oxis International, Portland, OR)] which donate NO^• ^its pure form into the aqueous environment. The other two compounds were thiol-based nitric oxide donors [(±)-S-Nitroso-N-acetypenicillamine (SNAP) and N-(β-D-Glucopyranosyl)-N^2^-acetyl-S-nitroso-D,L-penicillamide) glyco-SNAP (Oxis International, Portland, OR)], which preferentially donate NO^+^-equivalents to other thiols. Additionally, these donors differed in their stability. Based upon our spectrophotometric analysis at 37°C and a pH of 7.4, Spermine-NONOate has t^1/2 ^= 5 hours, DETA-NONOate a t^1/2 ^≈ 24 hours, SNAP a t^1/2 ^= 10 hours, and glyco-SNAP a t^1/2 ^= 28 hours [data not shown]. Furthermore, NONOate-based donors donate two molar equivalents of NO_X _per mole of donor compound, whereas the thiol donors deliver only one mole of NO_X _per mole of donor compound. These donor compounds were chosen to assess whether differences in the concentration, mode, or the rate of NO_X _delivery alter the genotoxicity of this radical species.

### Nitrite Production in Media

Nitric oxide donors were added to media without cells or to cell supernatants at increasing concentrations from 75 to 600 μM. After 24 hours of incubation the amount of nitrite produced in the media was assayed by the Greiss reaction as previously described [12,13]. Briefly, 50 μl of media was added to 50 μl of 1% sulfanilamide in 2.5% H_3_PO_4_. Then 50 μl of 0.1% napthylethylenediamine dihydrochloride in 2.5% H_3_PO_4 _was added in a 96-well microtiter plate. After incubation at room temperature for 30 minutes, the absorbance was measured on a microplate reader (Molecular Devices, Sunnyvale, CA) at 540 nm. The concentration of nitrite in the media was quantified as derived from standard curves created by adding known concentrations of NaNO_2 _from 50 to 300 μM sodium nitrite. NO_X _delivery kinetics was confirmed by determining changes in λ maximum of each compound over time on a spectrophotometer (Beckman Coulter DU530, Fullerton, CA).

### Single Cell Gel Electrophoresis (COMET assay)

The COMET assay was performed according to the procedure of Singh et al. with a few modifications [14]. Briefly, 120 μl of 0.5% normal melting point agarose in Ca^+2 ^and Mg^+2^-free phosphate buffer at 56°C were quickly layered onto a fully frosted slide and immediately covered with a cover-slip. The slides were kept at 4°C to allow the agarose to solidify. After gently removing the cover-slip a 50 μl aliquot of cell suspension was mixed with an equal volume of 1% low melting point agarose (Sigma, St. Louis, MO) at 37°C and quickly pipetted onto the first agarose layer in the same manner. Finally, 70 μl of 0.5% LMP agarose was added to cover the cell layer. The slide sandwiches without cover-slips were immersed in freshly prepared, cold lysing buffer [2.5 mol/l NaCl, 100 mmol/l Na_2_EDTA, 10 mmol/l Tris, 1% *N*-Lauroyl sarcosine sodium salt, pH 10, with 1% Triton X-100 added just before use] and kept at 4°C for 45 min to 1 hour.

The slides were placed on a horizontal gel electrophoresis platform and covered with cold alkaline buffer [300 mmol/l NaOH, and 1 mmol/l Na_2_EDTA] for 8 to 20 minutes in the dark at 4°C to allow DNA unwinding and expression of the alkali-labile sites. The timing for lysis and unwinding was determined empirically for each cell line. Electrophoresis was conducted at 4°C in the dark for 20 min at 25 V and 300 mA. The slides were then rinsed gently twice with neutralizing buffer (0.4 mol/l Tris, pH 7.5). Each slide was stained with 120 μl of propidium iodine (Sigma) at a concentration of 5 μg/ml and covered with a cover-slip.

COMET tail lengths were quantified as the distance from the centrum of the cell nucleus to the tip of the tail in pixel units, with the mean tail length being determined as the mean length of twelve tails.

### TUNEL Assay for Apoptosis

A terminal deoxynucleotidyl transferase (TdT)-mediated dUTP labeling (TUNEL) method was used for the detection of apoptotic cell death. For this purpose, the Apop Tag-Plus in-Situ Apoptosis Detection Kit (peroxidase) (Oncor, Gaithersburg, MD) was used. The staining was performed according to the manufacturer's recommended procedure. In brief, cell cultures of unexposed or exposed cells were initially treated with proteinase K (20 μg/ml) for 15 minutes at room temperature prior to using TdT to label the 3'-OH ends of DNA with digoxigenin-labeled nucleotides (1 hour incubation at 37°C). The slides were then treated with anti-digoxigenin antibody-peroxidase conjugate for 30 minutes at room temperature, stained with 3'-3 diaminobenzidine tetrahydrochloride (DAB) for 5 minutes to produce the characteristic brown color of positive cells, counterstained with hematoxylin, and mounted. Sections included in the kit were stained and served as positive controls. Consecutive oil immersion (100× objective) fields were counted on an Olympus BX40 microscope. A minimum of 1000 cells were counted and the apoptotic index was calculated as the percentage of staining cells. Cells were defined as apoptotic when the whole nuclear area was labeled or when occasional labeled globular bodies (apoptotic bodies) could be observed in the cytoplasm. Apoptosis found in the untreated cell cytospins at this time point were found to be 1%. Negative controls were cytospins of our cell lines in their logarithmic phase of growth supplemented with conditioned media and FCS. No apoptotic cells were demonstrated in these cytospins.

### Annexin V-FITC/propidium iodine FACS

Flow cytometric determination of apoptosis was performed using a commercially available (R&D Systems, Minneapolis, MN) Annexin V-FITC/propidium iodine apoptosis detection kit. Untreated and treated cells were collected after 24 hours of incubation by trypsinization and centrifugation at 500 × g for 5–10 minutes at room temperature. Cells were washed and resuspended in ice cold PBS and pelleted by centrifugation. Cells were then resuspended in Annexin V incubation reagent at a concentration of 1 × 10^6 ^cells/100 μl, and incubated in the dark for 15 minutes at room temperature. Binding buffer was then added to each sample. Samples were then analyzed within one hour by flow cytometry, and evaluated based on the percentage of the population of cells staining low or high for Annexin V (apoptotic cells) and propidium iodide (necrotic cells).

### Caspase Activity Assay

Activities of caspase-3 and -9 were determined using the corresponding caspase activity detection kits (R&D Systems, Minneapolis, MN) as described previously [15]. Briefly, 100 μg of total protein was added to 50 μl of reaction buffer, and 5 μl of substrates DEVD-pNA and LEDH-pNA were used to analyze the activity of caspase-3 and -9 respectively. Samples were incubated at 37°C for 3 hours and the enzyme-catalyzed release of pNA was quantified at 405 nm using a microtiter plate reader. The values of treated samples were normalized to corresponding untreated controls allowing determination of the fold increase or decrease in caspase activity. Alterations in enzymatic activity directly attributable to NO_X _were determined by the change in caspase activity in the presence or absence of 1 mM DTT.

### Cell Viability Assay

Cell viability was determined via a modified MTT assay as previously described [16]. In brief, cells were incubated with the various NO_X _donors for 24 hours at 37°C prior to cell viability assay. The media was aspirated and replaced with 0.2 ml of MTT (1 mg/ml in PBS), followed by incubation at 37°C for 5 hours. MTT was then aspirated and the wells were air dried for 5 minutes. The crystals were dissolved with 200 μl of DMSO, until the solution turned purple and absorbance analyzed in an enzyme-linked immunosorbent assay (ELISA) plate reader at 540 nm.

### Nitrotyrosine Staining

Pathologic nitric oxide exposure was confirmed by immunostaining using an anti-nitrotyrosine (Upstate Biotechnology, Lake Placid, NY) monoclonal antibody by immunoperoxidase as previously described [17]. The nitrotoyrosine Mab have previously demonstrated specific activity against human nitrotyrosine.

### Statistical Analysis

Statistical evaluation was performed using Prism-3.00 (GraphPad Software Inc., San Diego, CA). A p-value of <0.05 was considered significant. Values listed represented mean ± the standard error of the mean unless otherwise indicated.

## Results

### Increasing Concentrations of NO_X _donors Increases Nitrosative Stress

**Figure 1:**
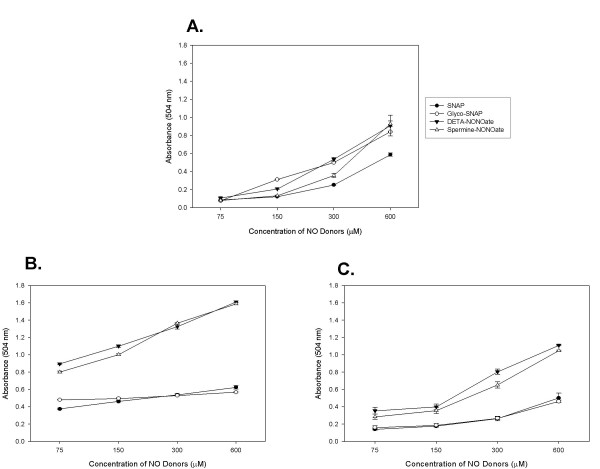
**Nitrite Production in Media and Supernatants in the Presence of Increasing Concentrations of NO_X _Donors: **A. Nitrite production in media without cells, B. Nitrite production in WI38 supernatants, C. Nitrite production in A549 Supernatants. Measurements of nitrite production in media alone or the supernatant of WI38 and A549 as measured by the Greiss reaction demonstrate increasing concentrations of nitrite production in the presence of increasing concentrations of NO_X _donors. This confirms that our NO_X _donors are liberating NO_X _in a concentration-dependent fashion. Interestingly, the curves for SNAP and glycol-SNAP were shifted toward the right in the supernatants of both WI38 and A549 possibly reflecting the possibility that these thiol-based NO_X _donors were preferentially donating their NO_X _to intracellular thiols.

Our first aim was to confirm that adding nitric oxide donors into aqueous media increased NO_X _production in a concentration dependent fashion. To investigate this, we determined the accumulation of NO_2_^- ^in the supernatants based on the Greiss reaction. Figure 1 demonstrates the concentration of nitrite in media in the absence of and in the presence of our two cell lines. In the absence of cells, we demonstrated an increasing concentration of nitrite within the media after 24 hours of incubation of various concentrations of all four NO_X _donors. This increase in nitrite accumulation was the same when NO_X _donors were added to the media alone or to the supernatants of our two cell lines. These data proved that our NO_X _donors were liberating NO_X _in a concentration dependent fashion.

We then confirmed that the published half-lives of our donor compounds were consistent with the kinetic release of NO_X _in our system. Using λ maximum specific for each donor compound, we found that the decay in absorbance to half-maximum roughly reflected the published t^1/2 ^for each of our donor compounds [data not shown].

To confirm that our cell lines were indeed being exposed to increasing amounts of NO_X _resulting from the addition of our NO_X _donors, we examined immunostaining for nitrotyrosine [Table 1]. In the absence of NO_X _donors, both cell lines demonstrated low levels of nitrotyrosine staining which increased with the addition of our donor compounds. The addition of exogenous NO_X _increased the staining intensity for nitrotyrosine in both cell lines, which appeared to saturate at a moderate level of immunostaining for nitrotyrosine. This result suggests ongoing low level of autologous nitration, and confirms that cells are being exposed to increasing nitrosative stress with the addition of NO_X _donors.

**Table 1:** 

**Cell Line**	**Nitrotyrosine Staining (Intensity)**					
	
	**NO^• ^Donor Concentration (μM)**	**Control**	**SNAP**	**Glyco-SNAP**	**DETA**	**Spermine**
**A549**	0	+				
	75		++	+	+	++
	150		++	++	++	++
	300		++	++	++	++
						
**WI38**	0	+				
	75		+	++	+	++
	150		+	++	++	++
	300		++	++	++	++

Mean nitrotyrosine staining intensity per 100 cells: 0+-no staining, 1+-mild staining, 2+-moderate staining, 3+-intense staining.

### DNA strand-breaks after exposure to NO_X_

**Figure 2:**
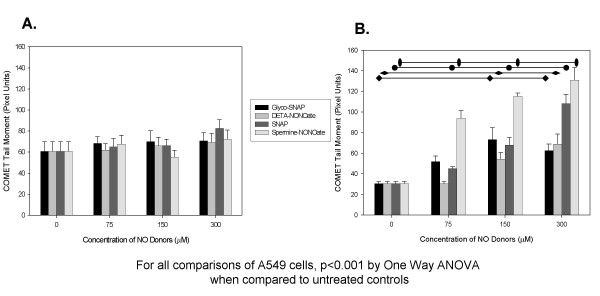
**COMET Tail Moment Lengths After 24 hour Exposure to Various NO_X _Donors: **A. COMET tail moment of WI38 after 24 hours of exposure to increasing concentrations of NO_X_, B. COMET tail moment of A549 after 24 hours of exposure to increasing concentrations of NO_X_. Measurements of the COMET tail moments for WI38 and A549 in the presence of increasing concentrations of NO_X _donors demonstrate that A549 was found to have increasing DNA strand breaks as nitrosative stress increased which was not seen in the WI38 control cell line. As seen in the graph, NO_X _donors with a long half-life [i.e. glyco-SNAP with a t^1/2 ^= 28 hours and DETA-NONOate with a t^1/2 ^of 20 hours] demonstrated significant increases in COMET tail moments only at the higher concentrations of NO donor, whereas NO donors with a shorter half-life [i.e. SNAP with a t^1/2 ^of 10 hours and Spermine-NONOate with a t^1/2 ^of 39 minutes] demonstrated significant increases in DNA strand breaks at lower concentrations. This confirms that A549 demonstrates more genomic instability in the presence of increasing nitrosative stress, which is not seen in control cells.

**Figure 3:**
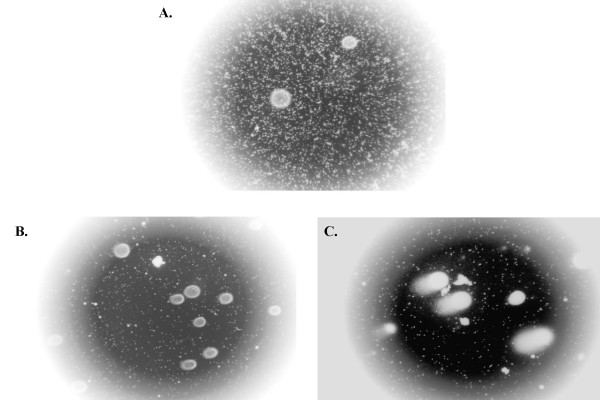
**Example COMET Tail Moments for A549: **These are example COMET tail moments for A549 in the presence of A.) 0 μM, B.) 75 μM, and C.) 150 μM Spermine-NONOate. Note that a significant increase in tail moment can be visualized with increasing concentrations of this NO_X _donor.

In order to quantify the ability of exogenous NO_X _to cause mutational events, we performed the in-vitro single-cell gel electrophoresis assay (the COMET assay). Increasing concentrations of these NO_X _donors were added to the supernatants of our cell lines and DNA strand-breaks were measured. As seen in Figure 2, distinctly different susceptibility to NO_X _exposure was seen in WI38 when compared to the tumor cell lines A549. Increasing concentrations of NO_X _donors increased DNA strand breaks in a concentration dependent fashion in the lung adenocarcinoma cell line, A549. Exposure of WI38 cells to increasing concentrations of NO_X _failed to cause an increase in DNA fragmentation that was detectable by the COMET assay. Examples of COMET tail moments seen in untreated A549 cells, in cells exposed to low concentrations, and high concentrations of NO_X _donors are seen in Figure 3.

### Determination of Cell Viability as a result of NO_X_-dependent DNA Fragmentation

**Figure 4:**
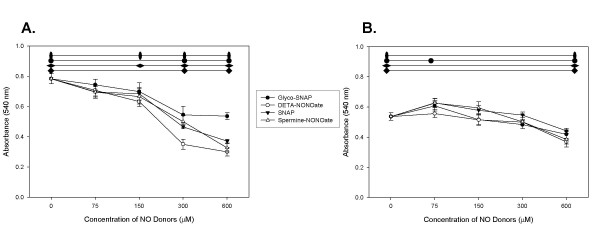
**Cell Viability as Determined by the MTT Assay with Exposure to Increasing Concentrations of NO_X _Donors: **A. Cell viability of WI38 as measured by the MTT assay after exposure to increasing concentrations of NO_X_, B. Cell viability of A549 as measured by the MTT assay after exposure to increasing concentrations of NO_X_. The cell viability was significantly reduced in WI38 the presence of increasing concentrations of NO_X _donors at all concentrations greater than 150 μM (p < 0.007 for all) for DETA- and spermine-NONOate, whereas the thiol-based glycol-SNAP and SNAP did not significantly decrease cell viability until 300 μM concentrations. These results are contrasted with A549 cells which did not demonstrate a significant decrease in cell viability (p < 0.03) until 600 μM concentrations of either thiol-based or NONOate-based NO_X _donors. Interestingly, the lowest concentration of SNAP (75 μM) demonstrated a significant growth advantage (p = 0.01).

We then utilized the MTT assay to explore if this NO_X_-donor compound induced DNA fragmentation caused a decrease in cell viability. As demonstrated in Figure 4, both cell lines were found to have a decrease in cell viability after exposure to increasing concentrations of either thiol based or NONOate-based NO_X _donors. Our control fibroblast cell line appeared to be more susceptible to NO_X _exposure, in that WI38 demonstrated a significant decrement in cell viability between 75 and 300 μM. Interestingly, the NONOate-based NO_X _donors were able to significantly decrease the cell viability of WI38 cells at a lower concentration than the thiol-based NO_X _donors. This is contrasted with our tumor cell line, A549. The decrement in cell viability for A549 was not significant until 600 μM of these NO_X _donors. In fact, a significant increase in cell growth was seen at the lowest concentration (75 μM) of SNAP in our A549 cells. Therefore, within the range of concentrations of NO_X _donor compounds studied, we have found that NO_X _has the ability to induce DNA fragmentation in A549 but not in the WI38 cells, while significantly decrease cell viability for WI38 relative to A549 cells.

### Determination of apoptosis after exposure to NO_X _donors

We then sought to determine whether NO_X_-mediated increases in DNA strand breaks correlated with apoptosis via the TUNEL assay. Since a decrease in cell viability was not seen until A549 cells were exposed to 600 μM NO_X_-donor, we examined apoptosis after exposure to this concentration. Cultured cells were exposed to 600 μM of the thiol-based NO_X_-donor, SNAP, or the NONO-ate based NO_X _donor, Spermine. Either NO_X _donor compound exposure for 24 hours did not significantly increase the percentage of TUNEL positive cells when compared with untreated controls [data not shown].

**Figure 5:**
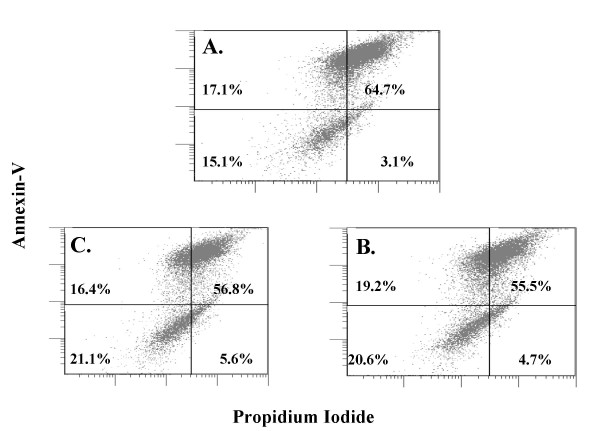
**Annexin-V/Propidium Iodine FACS in A549 Cells Unexposed and Exposed to High Concentrations of NO_X _Donors: **A. Untreated control, B. 600 μM Spermine NONOate treated, C. 600 μM SNAP treated. Annexin-V/Propidium Iodine FACS confirms the TUNEL results that demonstrate that no significant increase in apoptosis is noted in A549 with or without exposure to high nitrosative stress.

Because TUNEL positivity represents a late event in the apoptotic cascade, we wanted to ensure that the assay time frame did not represent assessment prior to detectable apoptosis. Therefore, we confirmed our apoptosis results via Annexin-V/propidium iodide FACS sorting. After exposure of our cell lines to 600 μM of SNAP or Spermine-NONOate, we confirmed the absence of any significant increase in apoptosis (Figure 5).

### Determination of caspase activity after NO_X _exposure or inhibition of NO_X _production

**Figure 6:**
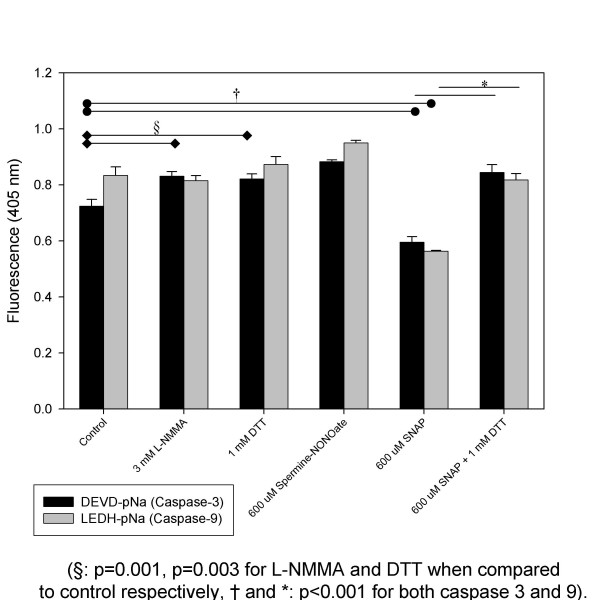
**Caspase 3 (DEVD-pNa) and Caspase 9 (LEDH-pNa) Activity as Measured by the in vitro Fluorogenic Caspase Assay in the Absence and Presence of High Concentrations of NO_X _Donors: **Both endogenous and exogenous NO_X _appears to influence caspase activity in A549 cells. Caspase 3 activity appears to demonstrate endogenous NO_X _inhibition since the addition of 3 mM L-NMMA or 1 mM DTT significantly increased caspase 3 activity (p = 0.001, p = 0.003 respectively). No similar endogenous inhibition was seen with caspase 9. The addition of the thiol-based NO_X _donor, SNAP (600 μM), significantly inhibited both caspase 3 and caspase 9 (p < 0.001 for both) when compared with control cell caspase activity. This caspase activity was significantly reversed with the addition of DTT (p < 0.001 for both caspase 3 and 9). Treatment groups: 1) Control, 2) 3 mM L-NMMA, 3) 1 mM DTT, 4) 600 μM Spermine-NONOate, 5) 600 μM SNAP, 6) 600 μM SNAP + 1 mM DTT.

In an attempt to explain these seemingly contradictory data, we examined the role of NO_X _in the modulation of caspase enzyme function via an in vitro caspase activity assay (figure 6). Using the artificial substrates DEVD-pNA and LEDH-pNA for assaying caspase-3 and -9 activity respectively, we demonstrated a basal inhibition of caspase-3 in A549 cells as demonstrated by a significant increase in caspase substrate cleavage with the addition of the NO_X_-specific inhibitor, N^Ω^-monomethyl-L-arginine (L-NMMA), when compared to control cells. This inhibition appears to be attributable to basal S-nitrosation of caspase-3, since the addition of 1 mM DTT also significantly increased caspase-3 activity. With the addition of exogenous NO_X _via our thiol-based donor compound, SNAP, a significant decrease in the activity of both caspases was found. In contrast, our NONOate-based NO_X _donor, Spermine, was unable to decrease either caspase-3 or -9 activities. The inhibition of both caspase's activity by SNAP was reversed with exposure to 1 mM DTT, suggesting that transnitrosation of these caspase enzymes may contribute to the inhibition of enzymatic activity. These data suggest that thiol-based NO_X_-mediated inhibition of caspase activity in A549 cells may cause a relative tolerance to ongoing DNA damage by inhibiting apoptosis.

## Discussion

We have demonstrated that NO_X _genotoxicity is highly dependent upon the concentration and kinetics of delivery of NO_X_. In addition, tumor cells appear to have increased genomic instability but increased resistance to ongoing NO_X_-mediated genotoxicity when compared to more normal transformed cells which demonstrate less genomic instability but increased susceptibility to ongoing DNA damage. Moreover, the mechanism of NO_X _delivery appears to have profound consequences upon the function of downstream effector molecules such as caspases.

Over the past 20–30 years key proteins have been increasingly implicated as targets for nitric oxide signaling. With respect to its role in cancer, both carcino-protective [18] and carcinogenic roles [19] have been attributed to NO_X_. The seemingly paradoxic nature of NO_X _in cancer has made investigations in this area quite enigmatic.

Increasing evidence supports NO_X_'s role in the induction and promotion of cancer [8,20]. Chronic inflammatory nitrosative stress has been implicated in carcinogenesis in a number of other organ systems [21,22]. Within this lung adenocarcinoma model, it is easy to hypothesize that either endogenous chronic inflammatory nitrosative stress as seen with idiopathic pulmonary fibrosis, or exogenous NO_X _as a result of cigarette smoking may predispose individuals to the development of this type of cancer.

One mechanism by which NO_X _has been hypothesized to be carcinogenic is through oxidative/nitrosative DNA damage and genotoxicity [23-25]. NO_X _can induce mutational events via DNA oxidation, deamination, point-mutations, and strand-breaks, thus contributing to the multi-step process of carcinogenesis. A number of papers have documented that NO_X _can cause DNA strand breaks in areas of chronic nitrosative stress [26-28]. Our data demonstrates that in the human lung adenocarcinoma cell line, A549, increasing concentrations of NO_X _donors increases DNA strand-breaks in a concentration dependent fashion. Moreover, the kinetics of NO_X _exposure also affects its genotoxicity. It is hypothesized that this ongoing DNA damage can lead to cancer development. In support of this notion, in a mouse model of inflammatory mediated lung carcinogenesis, studies have demonstrated that genetic ablation of the inducible isoform of nitric oxide synthase decreased the lung tumor multiplicity after exposure to the carcinogen, urethane [29].

The data presented herein of NO_X_-dependent DNA strand breaks in A549 cells is in stark contrast to NO_X_'s inability to induce DNA fragmentation in our control fibroblast cell line, WI38. Normally, DNA damage is repaired by a complex series of DNA repair mechanisms. Defects in repair mechanisms can predispose people to cancer development [30]. An increasing body of evidence supports epigenetic modulation of enzymatic function, such as S-nitrosation [31]. As a result of nitrosation of key thiol groups within DNA repair enzymes, DNA-damage repair can be inhibited potentially perpetuating mutational events. One possible explanation for these opposing sets of data is that A549 may be capable of expressing unstimulated NOS activity [32], and therefore may have saturated such intracellular thiols as glutathione. In support of this hypothesis, immunostaining for nitrotyrosine in both cell lines found low levels of nitrotyrosine staining in the absence of exogenous NO_X_. Research is ongoing to determine whether tumor elicited NO_X _production may contribute to tumor progression.

If DNA damage overwhelms repair mechanisms, normal cells are triggered to undergo apoptosis. Redundant mechanisms for the induction of apoptosis exist, but the most common pathway involves the induction of p53. With the induction of p53, apoptosis is triggered through the release of cytochrome c and caspase activation. Despite possessing wild-type p53, our data demonstrated that in A549 cells we were unable to detect a decrease in cell viability as measured by the MTT assay until very high concentrations of donors, or an increase in cell apoptosis via either the TUNEL assay or Annexin V/propidium iodide FACS. There are conflicting results in the literature regarding the effects of exogenous NO_X _on cell viability and apoptosis. Certain studies have shown that NO_X _by itself does not cause A549 cells to die, but the addition of hyperoxia induces rapid cell death [33], perhaps through the production of peroxynitrite. On the other hand, other studies have demonstrated that the addition of the NO_X _donor SNAP decreased cell viability via apoptosis in a concentration dependent fashion [34]. This study went on to analyze apoptosis in cells other than A549, thus precluding a direct correlation with A549 and apoptosis after SNAP exposure.

Further confusing the interrelationship between DNA fragmentation and apoptosis is the fact that the prevailing opinion in the literature is that there exists a direct correlation between the fragmentation seen in the COMET assay and the fragmentation seen in apoptosis. With triggering of apoptosis, DNA strands are cleaved or nicked by nucleases, exposing 3'-hydroxyl ends. Nick-end cleavage may not necessarily equate with strand breaks seen with various mutagens. A growing body of evidence suggests that the fragmentation found in the COMET assay is not necessarily related to apoptotic fragmentation [35,36]. Studies have demonstrated that COMET tail moments of cells undergoing apoptosis are highly fragmented to the point of loosing nuclear architecture [37]. Furthermore, nitroso-compound related DNA damage appears to be independent of more commonly accepted measures of apoptosis, such as the TUNEL assay [38]. The mechanistic basis for these disparate measures remains to be determined, but appears to represent fundamental differences in the characteristics of exposed 3' ends seen with oxidative DNA damage versus apoptosis related DNA cleavage. Therefore, the COMET assay may be a measure of genotoxicity, without necessarily detecting apoptosis.

Lending credence to NO_X_'s possible carcinogenic role is the implication from several lines of study that NO_X _can influence enzymatic function of such enzymes as caspases [11]. In this way, NO_X _may increase the threshold by which cells that have undergone a level of genotoxicity would be triggered to undergo apoptosis. If any member of the apoptotic pathway is lost or inhibited, the threshold for apoptosis would theoretically be increased. NO_X _has demonstrated a capability of inhibiting caspase activity through S-nitrosation of key thiols [15,39]. Our data supports the NO_X_-mediated inhibition of both caspase-3 and -9 in A549 cells with ongoing DNA mutational events. In fact, prior data has demonstrated that basal inhibition of mitochondrial caspases by cysteine nitrosation must be removed in order to activate the caspase-mediated apoptotic pathways [40]. Our data supports this in A549 in that the addition of the antioxidant, DTT, increased caspase activity detectable by our in fluorogenic assay. One potential mechanism for this inhibition of casapase activity is the S-nitrosation of essential thiol groups [40]. The fact that our thiol-based NO_X _donor appeared to inhibit caspase activity more efficiently that the NONOate donor further supports this notion, since it is well known that nitroso-thiols will preferentially transnitrosate other thiols [41]. In light of the increasing interest in NO_X_-donor compounds as cytotoxic cancer therapy, a careful consideration of potential downstream effects of the mode of NO_X _delivery should be undertaken. Caspases can be inhibited not only by direct protein S-nitrosation, but also indirectly by a cGMP-mediated pathways [39]. The return of caspase activity with the addition of DTT would further suggest that caspase inactivation was mediated by S-nitrosation in our lung adenocarcinoma model.

The implications of these data can be applied both to the understanding of tumor progression as well as the design of NO_X_-based chemotherapeutic strategies. It is well established that tumors demonstrate a high rate of cellular turnover, with the vast majority of cells undergoing cell death resulting from ongoing genomic instability. Only those clones possessing a survival advantage will be able to repopulate the tumor and thus contribute to increasing aggressiveness, the ability to invade and metastasize, and resistance to further therapeutic interventions. Chronic nitrosative stress may be contributing to this process by selecting for more virulent clones via inducing DNA mutational events and perpetuating these mutations through a relative inhibition of apoptosis. Moreover, as more investigations explore the potential use of NO_X _delivery as a possible cytotoxic chemotherapy, the issues of concentration, kinetics, and mechanism of delivery must be carefully considered in order to avoid untoward pro-carcinogenic side effects.

## Conclusions

In conclusion, we demonstrate that tumor cells experience an increase in DNA strand breaks with increasing nitrosative stress relative to the concentration and kinetics of delivery. This nitrosative stress does not correlate with increased cell death or apoptosis in established tumor cell lines, which is in stark contrast to non-tumor immortalized cells. The lack of apoptosis associated with increased DNA fragmentation may in part be explained by the inhibition of caspase-3 and -9 activity by thiol-based delivery but not NONOate-based delivery of NO_X_. Taken together, these data support the role of NO_X _in nitrosative genomic instability as well as inhibiting apoptosis, implicating it in cancer promotion. The demonstration of these same findings in rodent cell lines would establish the foundation for animal models with which to fully elucidate the role of NO_X _in tumor development and growth.

## Author's contributions

BB conceived of the study, participated in the design of the study, and performed the statistical analysis. GH interpreted the results of the FACS and immunoassays. NH carried out the majority of the COMET assays, as well as performing the caspase assay. JR participated in the study design and coordination. All authors read and approved the final manuscript.
